# Supplementary material for: Phenotypic Switching of Naïve T Cells to Immune-Suppressive Treg-Like Cells by Mutant KRAS
Source: J Clin Med. 2019 Oct 18;8(10):1726. doi: 10.3390/jcm8101726 (PMC6832522; doi:10.3390/jcm8101726)
Supplement: Supplementary file 1 [file jcm-08-01726-s001.pdf]

Supplementary Materials

Model I:

The set of equations for model I are given by:

$$\frac{dX}{dt} = \alpha X - \beta X - \beta_{XY} X$$

$$\frac{dY}{dt} = \alpha' Y - \beta' Y + \beta_{XY} X$$

where α and α' are respective proliferation rates, and β and β' are respective rates of cell death (apoptosis). β_{XY} denotes the irreversible transition rate from X ($CD4^+$) to Y (Tregs) due to a mutational mechanism.

Steady-state analysis of this model reveals that either X=0, Y=0; or that $\alpha = \beta + \beta_{XY}$. $CD4^+$ T cells have been shown to have long half-lives (of the order of a few months) (1), but they proliferate at a much faster rate (2). Given that the integration of mutated KRAS DNA into DNA of Tregs is expected to be infrequent (based on likelihood of successful integration), $\alpha > \beta + \beta_{XY}$. Thus, as per this model, $\frac{dX}{dt} > 0$, hence the population of Tregs cells—given by $Y = \frac{\beta_{XY} X}{-\alpha' + \beta'}$ —will most likely continue to grow.

Model II:

The set of equations for model II is given by:

$$\frac{dX}{dt} = \alpha X - \beta X$$

$$\frac{dY}{dt} = \alpha' Y - \beta' Y$$

where α and α' are respective proliferation rates, and β and β' are respective rates of cell death (apoptosis).

Due to the reasons discussed above in model I, the population of Tregs cells—given by $Y = Y_0 \mathrm{e}^{(\alpha' - \beta')t}$,where Y_0 is its initial number/concentration—is also expected to continue growing.

Model III:

The set of equations for model III is given by:

$$\frac{dX}{dt} = \alpha X - \beta X - \beta_{XY} X + \beta_{YX} Y$$
$$\frac{dY}{dt} = \alpha' Y - \beta' Y + \beta_{XY} X - \beta_{YX} Y$$

where α and α' are respective proliferation rates, and β and β' are respective rates of cell death (apoptosis). β_{XY} and β_{YX} are respective interconversion rates (phenotypic plasticity)

For steady state solution, $X = \frac{\beta_{YX} Y}{\beta_{XY} + \beta - \alpha}$ and $Y = \frac{\beta_{XY} X}{\beta_{YX} + \beta' - \alpha'}$.

These equations imply: $\frac{\beta_{YX}}{\beta_{XY} + \beta - \alpha} = \frac{\beta_{YX} + \beta' - \alpha'}{\beta_{XY}}$.

Based on the relatively quick transcriptional response involved in phenotypic plasticity, it is possible that a set of parameters can satisfy this condition, thus leading to a steady-state solution, i.e., the number of Treg cells plateau over time.

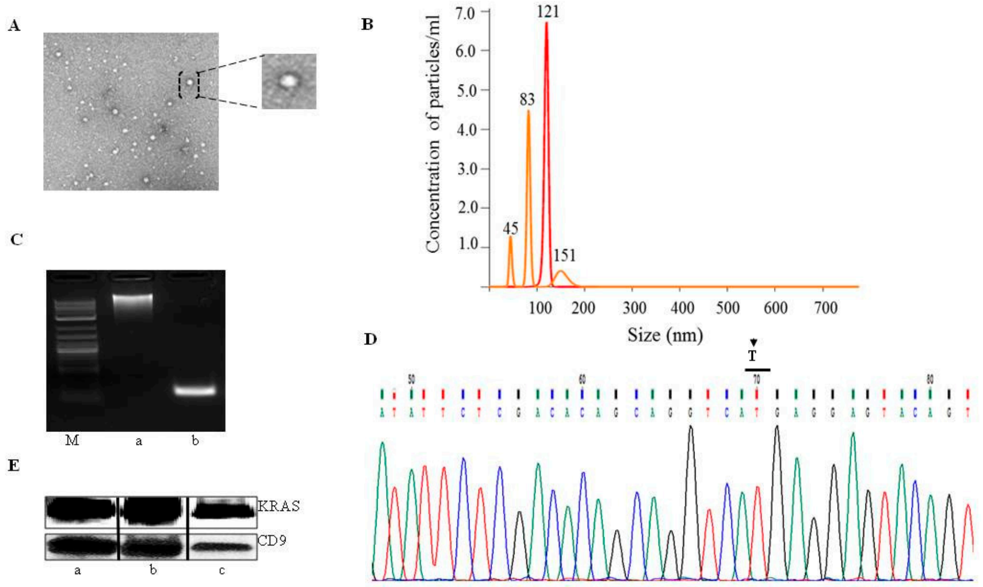

Figure S1. Isolation and characterization of tumor-derived exosomes (TDEs). (**A**) Transmission Electron Microscopy of isolated TDEs, and (**B**) Nanosight method to determine the concentration/mL and size (nm) of TDEs based on Brownian movement of the particles. (**C**) Exosomal DNA was run on 1.5% agarose gel. a) High-molecular-weight DNA corresponding to the genomic DNA. b) PCR-amplified DNA corresponding to exon 3 for codon 61-point mutation in KRAS. The amplified PCR product was subjected to Sanger sequencing. M) DNA size markers. (**D**) Chromatogram obtained from the Sanger sequencing for Q61H (A>T). (**E**) Immunoblot analysis of KRAS and the exosomal marker CD9 using TDEs isolated from a) A549, b) H460, and c) H1299 NSCLC cell lines. TDE particles were isolated from serum-free media from NSCLC cells. A total number of $1x10^6$ particles were used in each experiment.

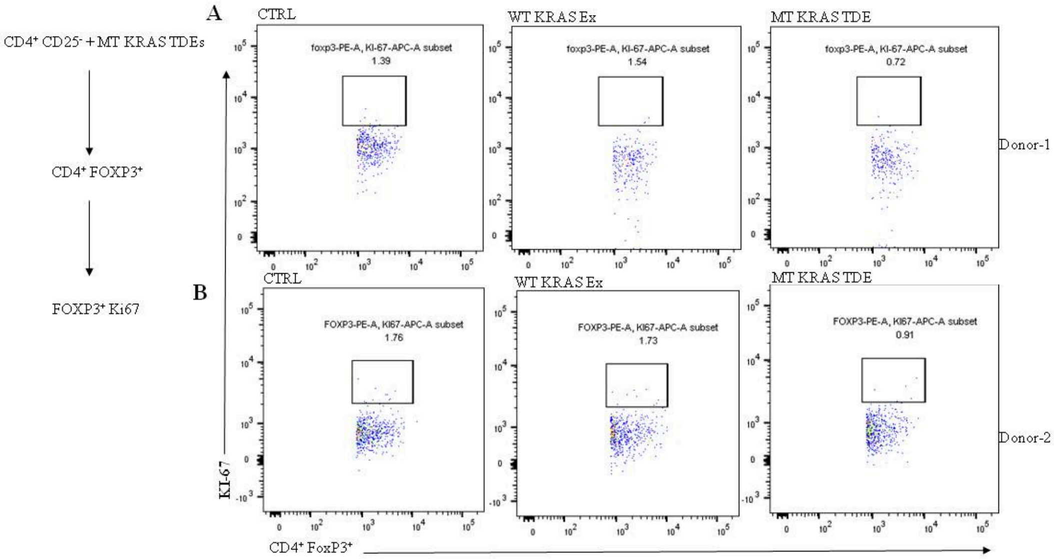

Figure S2. Analysis of FOXP3⁺ Treg cell proliferation using the Ki67 marker. Naïve CD4⁺ CD25⁻ T cells at a density of 1×10^6 cells were incubated with MT KRAS or WT KRAS TDEs, and then analyzed by flow cytometry for CD4⁺ FOXP3⁺ Tregs. The subpopulation of CD4⁺ and FOXP3⁺ Tregs was further gated for Ki67⁺ FOXP3⁺ expression. (**A**) and (**B**) Cells incubated with MT KRAS TDEs. No significant increase in expression of Ki67 compared to control or WT KRAS TDEs was observed.

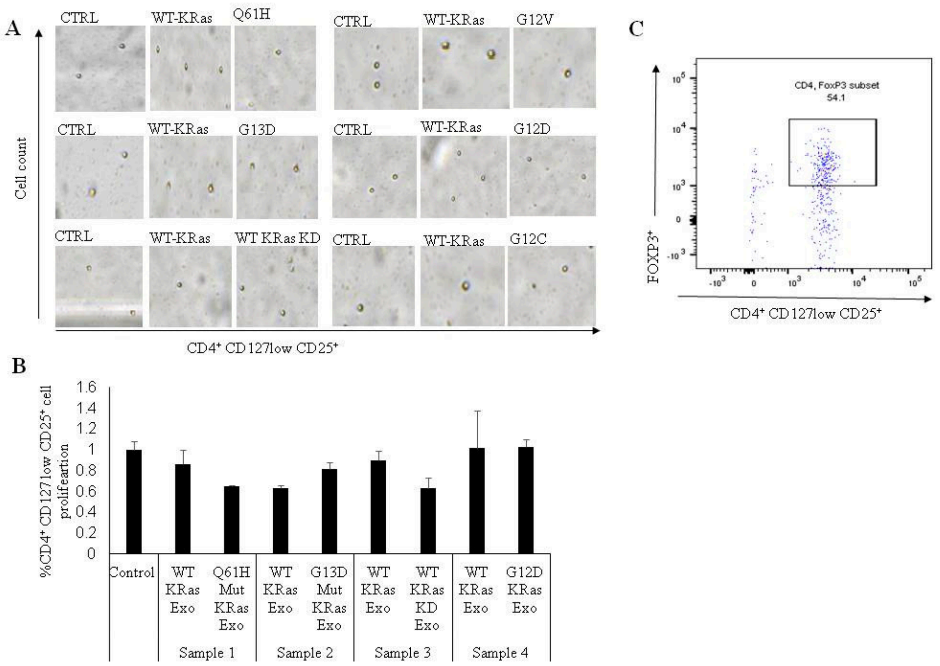

Figure S3. Analysis of CD4⁺ CD127^{low} CD25⁺ Treg cell proliferation. (**A**) CD4⁺ CD127^{low} CD25⁺ Tregs were incubated with NSCLC TDEs carrying KRAS mutations (G12D, G12V, Q61H, G13D, or G12C) or WT KRAS. After 24–48 h, live cells were counted using the Nexcelom Cellometer. Images for each KRAS mutation and WT KRAS population are presented. (**B**) The Tregs at a cell density of 1×10^4 cells/well were incubated with NSCLC MT KRAS TDEs and counted by using the IncuCyte Live Cell Imaging System. The bar chart depicts the mean and standard deviation (SD) of three independent experiments. The NSCLC MT KRAS serum-derived TDEs incubated with Tregs did not cause any increase in cell number as compared to control. (**C**) The dot plot depicts the isolated Tregs that were analyzed by flow cytometry for the percent FOXP3 expression by gating for CD4 FOXP3.

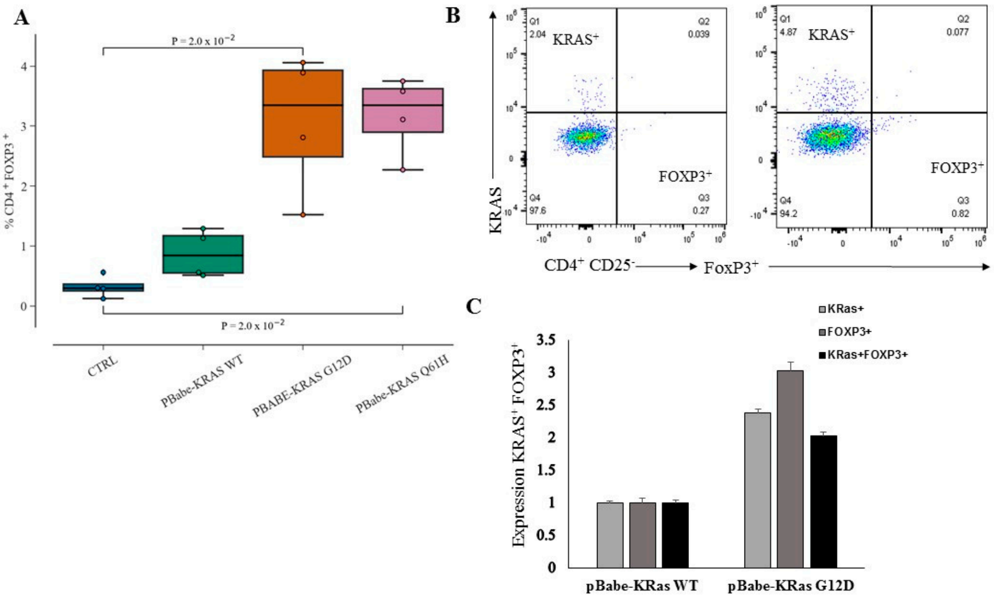

Figure S4. Immune phenotype conversion mediated by MT KRAS cDNA. (**A**) Naïve $CD4^+$ $CD25^-$ T cells were isolated from four independent donor PBMC by negative selection, as described in the Experimental Section. The naïve $CD4^+$ $CD25^-$ T cells at a density of 2×10^6/well were transfected with plasmid PBabe-KRAS WT or PBabe-KRAS G12D or PBabe-KRAS Q61H MT in a 24-well plate for 48 h, and then analyzed for $CD4^+$ and $FOXP3^+$ Tregs. The PBabe-KRAS G12D or PBabe-KRAS Q61H MT transfected cells showed a ~3-fold increase in $CD4^+$ $FOXP3^+$ Treg-like cells ($p=2\times10^{-2}$) compared to the control (CTRL). (**B**) and (**C**) TDEs isolated from the MT KRAS NSCLC lines were analyzed by NxT Attune flow cytometer for IL-10 cytokine expression. TDEs were isolated from MT and WT KRAS cells, diluted with 10-fold PBS, and analyzed for expression of the exosomal marker CD9 using a PE anti-human CD9. The levels of cytokines in the TDEs were determined using PE/CY7 anti-human IL-10 and Brilliant violet 428 anti-human TGF-β1 antibodies.

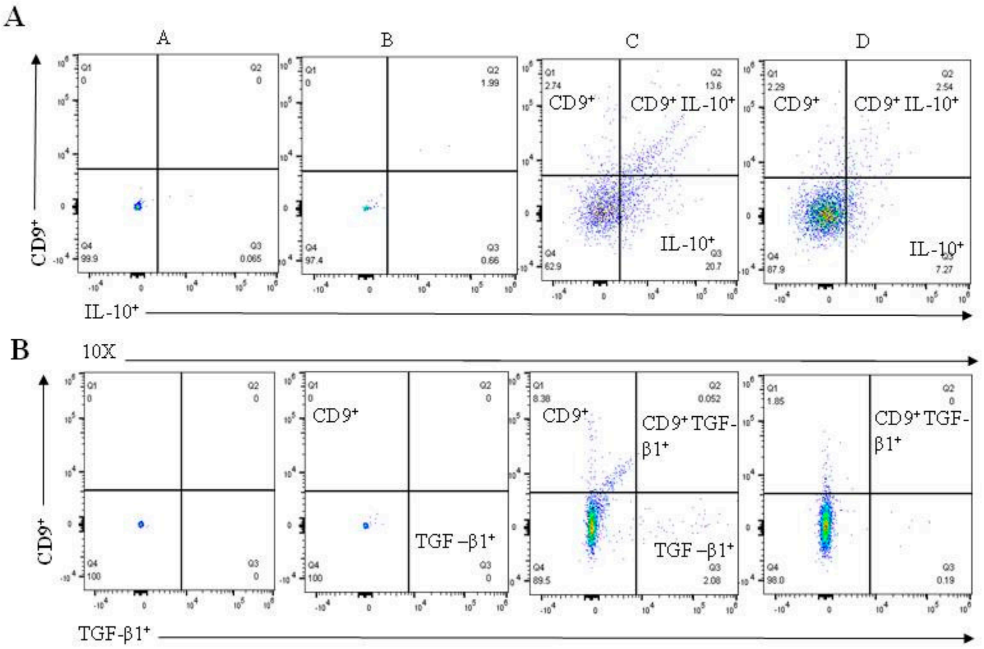

Figure S5. Immune phenotype conversion is not dependent on IL-10 or TGF- 1. The data are shown as dot plots obtained from the flow cytometry analysis for IL-10 and TGFβ1. Indicated in the dot plot a) PBS, b) negative control, c) WT KRAS TDEs, and d) MT KRAS TDEs. MT KRAS TDEs did not show any significant increase in IL-10 (**A**) or TGF-β1 (**B**) expression compared to WT KRAS TDEs.

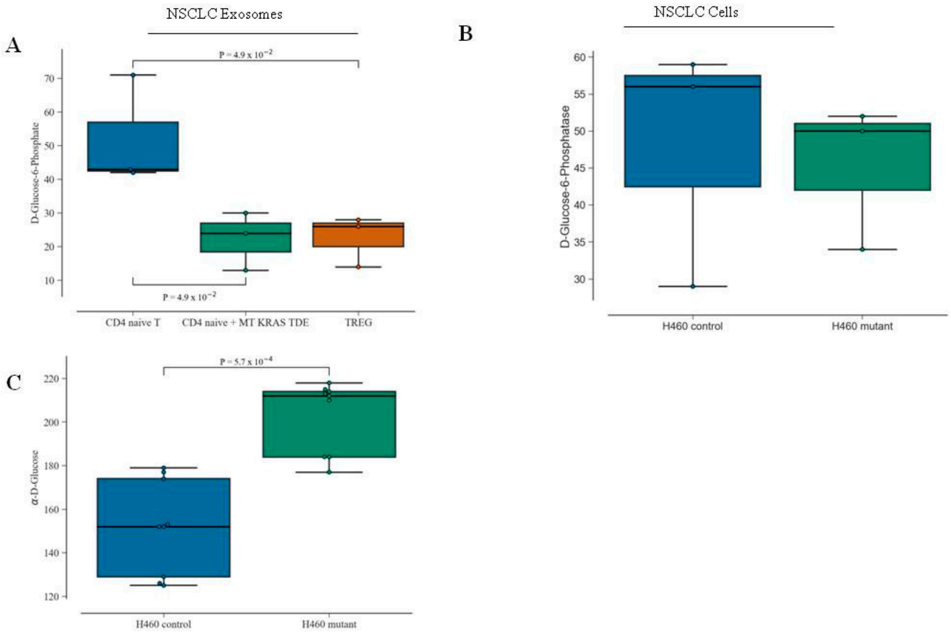

Figure S6. Metabolic profiling. Naïve CD4+ CD25- T cells induced to convert to Treg-like cells upon co-culture with TDEs were assayed using a pre-arrayed 96-well plate from Biolog. TDEs from MT KRAS NSCLC cells were seeded 20,000/well (12 wells each) and incubated with naïve CD4+ CD25- T cells. Naïve CD4+ CD25- T cells alone were used as a negative control, and CD4+ CD127^{low} CD25+ Treg cells were used as a positive control. The plates were incubated in an OmniLog incubator for 24 h. (**A**) Glucose-6-phosphate uptake was significantly higher in the CD4+ CD25- naïve T cells (p=4.9×10^{-2}) compared to MT KRAS TDEs. The latter had a level of redox potential similar to that of isolated CD4+ CD127^{low} CD25+ Treg cells, indicating that a low-glucose environment is required to sustain Treg-like cells. (**B**) Box plot for MT and WT KRAS NSCLC cells analyzed for glucose-6-phosphate utilization using the Biolog system. A significant reduction in MT KRAS cells was seen compared to WT KRAS cells. (**C**) A comparison D-glucose utilization by WT KRAS cells and MT KRAS cells. The box plot shows an increase in D-glucose (p=5.7×10^{-4}) compared to WT KRAS cells. The *p values* were calculated using the Mann–Whitney U-Test for statistical significance.

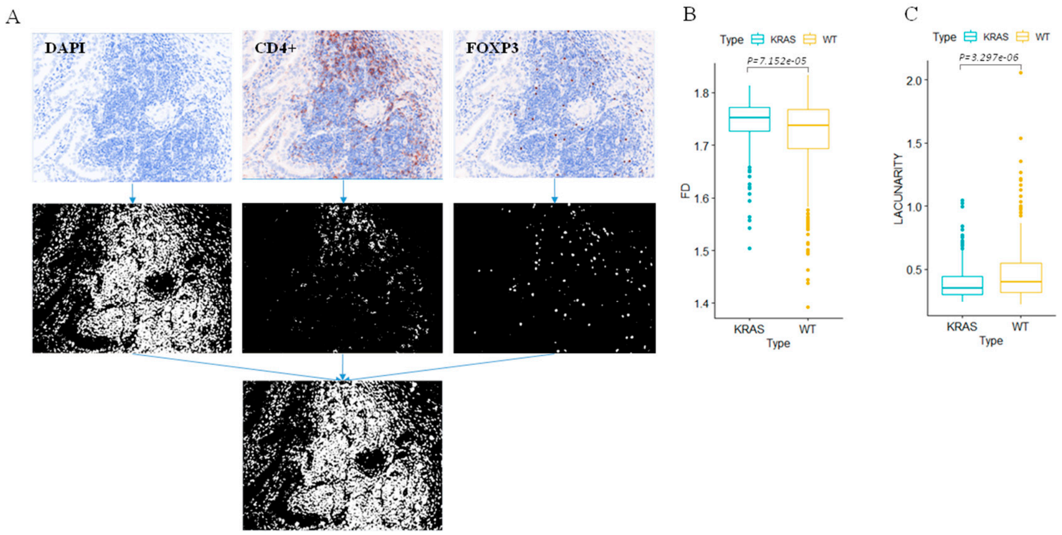

Figure S7. Fractal analysis performed on IHC images from tumor tissue from NSCLC patients with MT KRAS confirms that the tumor microenvironment is enriched in Tregs. (**A**) Representative images of immunohistochemical staining of DAPI, CD4, and FOXP3 of lung cancer patient tissue. The images for each marker were segmented using the Otsu threshold and converted to a binary image. The binary images for all three markers were merged for fractal analysis. (**B**) The distribution of fractal dimension of merged IHC images shows an increase of fractal dimension in KRAS mutant samples (p=7.152e-05). (**C**) The distribution of lacunarity of merged images shows a decrease of lacunarity in KRAS mutant samples (p=3.297e-06). The *p values* were calculated using the Mann–Whitney U-Test for statistical significance.

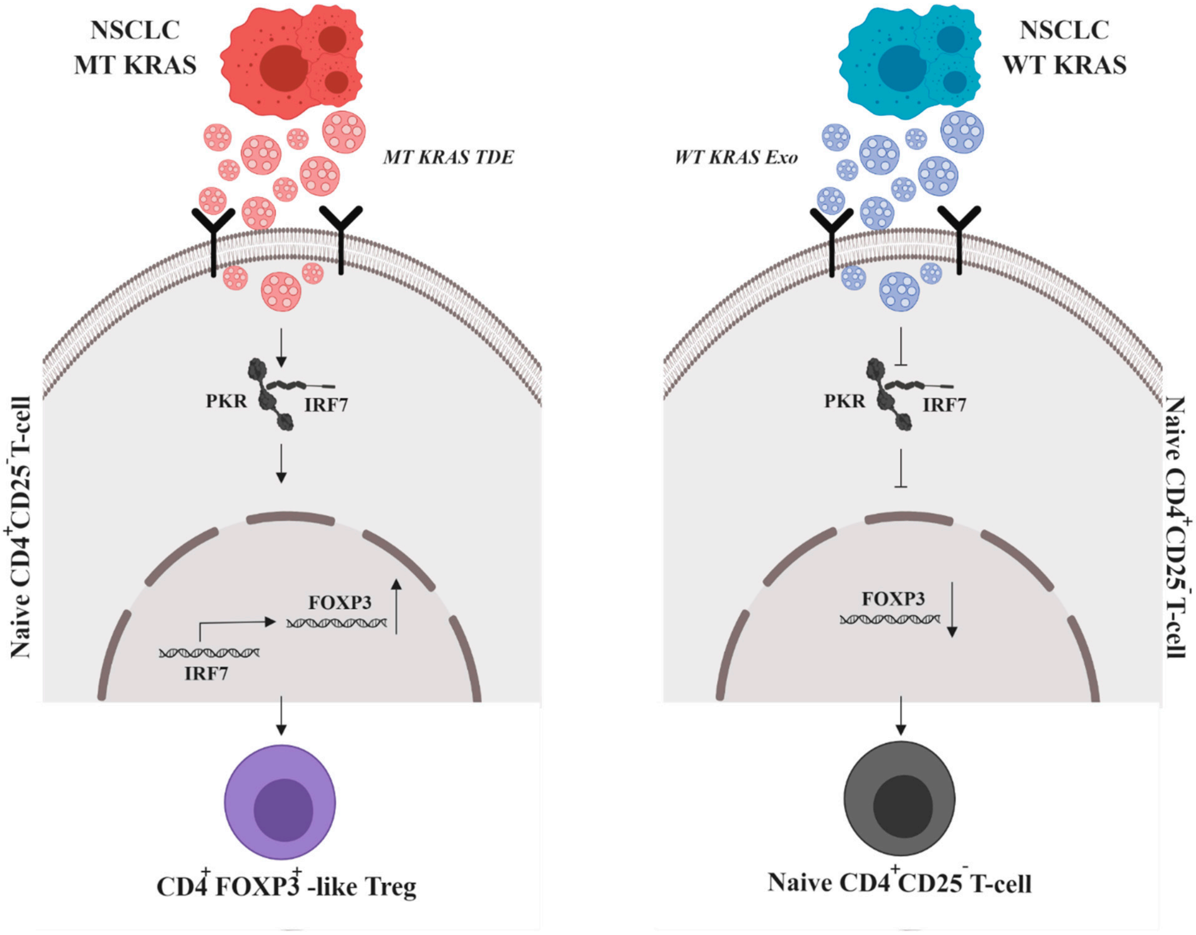

Figure S8. A model explaining the interferon pathway-mediated mechanism underlying MT KRAS-induced phenotypic switching in T cells. TDEs shed from NSCLC cells carrying mutant MT KRAS are incorporated into $CD4^+$ naïve T cells and induce the IRF7 signaling pathway. This leads to increased gene expression and induction of proteins involved in expression and secretion of type I IFN. Type I IFN, in turn, induces the conversion of $CD4^+$ naïve T cells to Treg-like cells by upregulating genes that characterize Tregs, including FOXP3. However, the switch is not a binary event, but a gradual process that appears to involve a series of bifurcations. Consequently, MT KRAS-mediated conversion yields a heterogeneous population with varying Treg-like phenotypes, including some that bear a close resemblance to bona fide Tregs, expressing the full complement of Treg-restricted genes, such as FOXP3.

Table S1. KRas codon 12, 61 PCR amplification and sequencing primers, Annealing temperature and Amplicon Size.

PCR and Sequencing	5′-3′ Sequence	Annealing Temperature (˚C)	Amplicon Size (bp)
KRas F	AAGGCCTGCTGAAAATGACT	59	170
KRas R	AGAATGGTCCTGCACCAGTAA	59	170
KRas 61F	TGCACTGTAATAATCCAGACTGTG	56	300
KRas 61R	GCATGGCATTAGCAAAGAC	56	300

Table S2. Metabolic profile for naïve CD4 T cells, naïve CD4 T cells incubated with TDEs, and bona fide Tregs.

**Hours**	**Source**	**Naïve CD4 T cell**	**Naïve CD4 +MTKRAS TDE**	**Treg**
24 hrs	**Dextrin**	16	48	28
36 hrs	**Dextrin**	0	56	45
48 hrs	**Dextrin**	13	63	48
72 hrs	**Dextrin**	15	71	60
24 hrs	**D-Glucose-6-Phosphate**	43	24	28
36 hrs	**D-Glucose-6-Phosphate**	30	24	26
48 hrs	**D-Glucose-6-Phosphate**	38	28	37
72 hrs	**D-Glucose-6-Phosphate**	44	39	40
24 hrs	**D-Glucuronic acid**	52	16	3
36 hrs	**D-Glucuronic acid**	58	28	21
48 hrs	**D-Glucuronic acid**	88	30	31
72 hrs	**D-Glucuronic acid**	100	47	53

24 hrs	**Palatinose**	0	41	31
36 hrs	**Palatinose**	0	42	69
48 hrs	**Palatinose**	0	0	91
72 hrs	**Palatinose**	15	11	116
24 hrs	**Adonitol**	12	13	0
36 hrs	**Adonitol**	29	5	0
48 hrs	**Adonitol**	37	6	0
72 hrs	**Adonitol**	33	6	0
24 hrs	**Citric acid**	28	0	0
36 hrs	**Citric acid**	10	0	0
48 hrs	**Citric acid**	30	0	0
72 hrs	**Citric acid**	28	0	0
24 hrs	**Tricarballylic acid**	28	0	0
36 hrs	**Tricarballylic acid**	12	0	0
48 hrs	**Tricarballylic acid**	23	0	0
72 hrs	**Tricarballylic acid**	12	0	0
24 hrs	**a-Keto-Glutaric acid**	41	18	30
36 hrs	**a-Keto-Glutaric acid**	32	23	25
48 hrs	**a-Keto-Glutaric acid**	45	29	22
72 hrs	**a-Keto-Glutaric acid**	50	36	34
24 hrs	**Succinamic acid**	0	0	0
36 hrs	**Succinamic acid**	21	0	0
48 hrs	**Succinamic acid**	32	0	0

72 hrs	**Succinamic acid**	18	0	0
24 hrs	**Acetoacetic acid**	64	38	41
36 hrs	**Acetoacetic acid**	29	29	23
48 hrs	**Acetoacetic acid**	45	28	32
72 hrs	**Acetoacetic acid**	31	24	28
24 hrs	**a-Keto-Butyric acid**	0	30	17
36 hrs	**a-Keto-Butyric acid**	0	14	16
48 hrs	**a-Keto-Butyric acid**	0	5	16
72 hrs	**a-Keto-Butyric acid**	0	6	25
24 hrs	**a-Hydroxy-Butyric acid**	29	18	0
36 hrs	**a-Hydroxy-Butyric acid**	40	5	0
48 hrs	**a-Hydroxy-Butyric acid**	42	0	0
72 hrs	**a-Hydroxy-Butyric acid**	52	0	2

Table S3. Common genes expressed in CD4 naïve T cells incubated with MT KRAS Exo and Tregs.

**p_val**	**avg_logFC**	**pct.1**	**pct.2**	**p_val_adj**	**cluster**	**gene**
1.30E-71	0.621962	0.735	0.375	1.37E-67	MT_KRAS_EXO	LY6E
0.004002	0.389766	0.537	0.528	1	Tregs.plus	LY6E
9.22E-54	0.617589	0.494	0.186	9.70E-50	MT_KRAS_EXO	EIF2AK2
0.002004	0.320215	0.379	0.307	1	Tregs.plus	EIF2AK2
1.48E-42	0.522276	0.425	0.164	1.56E-38	MT_KRAS_EXO	IRF7
0.000253	0.396483	0.356	0.263	1	Tregs.plus	IRF7
8.42E-33	0.388904	0.759	0.543	8.86E-29	MT_KRAS_EXO	RNF213
1.38E-09	0.527333	0.696	0.626	1.45E-05	Tregs.plus	RNF213
1.36E-28	0.387601	0.618	0.363	1.43E-24	MT_KRAS_EXO	EPSTI1

3.09E-22	0.692072	0.663	0.441	3.25E-18	Tregs.plus	EPSTI1
2.79E-22	0.364487	0.516	0.309	2.93E-18	MT_KRAS_EXO	SP100
2.88E-06	0.37878	0.492	0.382	0.030327561	Tregs.plus	SP100
4.46E-22	0.36649	0.453	0.254	4.70E-18	MT_KRAS_EXO	BST2
2.09E-05	0.342897	0.434	0.324	0.219813474	Tregs.plus	BST2
